# Self-generated thoughts and depression: from daydreaming to depressive symptoms

**DOI:** 10.3389/fnhum.2014.00131

**Published:** 2014-03-18

**Authors:** Igor Marchetti, Eowyn Van de Putte, Ernst H. W. Koster

**Affiliations:** Department of Experimental Clinical and Health Psychology, Ghent UniversityGhent, Belgium

**Keywords:** self-generated thought, daydreaming, mindwandering, self-focus, rumination, depressive symptoms, mindfulness, Default Mode Network

## Abstract

Human minds often engage in thoughts and feelings that are self-generated rather than stimulus-dependent, such as daydreaming. Recent research suggests that under certain circumstances, daydreaming is associated with adverse effects on cognition and affect. Based on recent literature about the influence of resting mind in relation to rumination and depression, this questionnaire study investigated mechanisms linking daydreaming to depressive symptoms. Specifically, an indirect effect model was tested in which daydreaming influences depressive symptoms through enhancing self-focus and ruminative thought. Results were in line with the hypothesis and several alternative pathways were ruled out. The results provide initial supportive evidence that daydreaming can influence depressive symptoms through influences on self-focus and rumination. Further research should use prospective or experimental designs to further validate and strengthen these conclusions.

All the daydreams are ego-centred.[…]

Each of the daydreams is like a little play, whose hero is the dreamer himself

G. H. Green (1923, pp. 27–28)

## Introduction

It often happens that one's mind drifts away from what one is doing or that one's attention fluctuates inward during rest. For instance, in everyday life it is a common experience, while reading a book, not to be able to recall the last page, or during a long train ride, to spend a large part of time being unaware of the other passengers. In other words, there is ample evidence that human mind often focuses on mental contents arising independently from the direct environment or the task at hand (Smallwood and Schooler, 2006; Smallwood, 2013). Consequently, this specific process has been defined “self-generated thought” (SGT; Smallwood, 2013).

Throughout decades, SGT has been operationalized in different ways (Callard et al., 2013), by focusing either on its independence from the ongoing task (i.e., stimulus-independent thought; Mason et al., 2007) or the internal rather than external source of information (i.e., spontaneous thought; Christoff et al., 2011). We here choose to operationalize SGT as “daydreaming” (Klinger, 2013), which is a comprehensive phenomenon that, beyond (i) being based on SGT, gathers all the mental states sharing crucial characteristics, such as (ii) the same neurobiological substrate (Stawarczyk et al., 2011), and (iii) similar subjective content (Smallwood and Schooler, 2006). By doing so, we have multiple advantages, such as being allowed to capitalize on previous literature (Klinger, 1971, 1990, 2009, 2013) and the related instruments of measurement (Singer and Antrobus, 1970, 1972; Giambra, 1980).

Daydreaming is conceived as “*nonworking thought that is either spontaneous or fanciful*” (Klinger, 2009, p. 226) and it is considered the default mode of the mind (Klinger, 1971; Mason et al., 2007). This definition includes SGT unrelated to the task at hand, also known as mindwandering (Smallwood and Schooler, 2006), as well as instances when the mind wanders toward fanciful topics during rest (Klinger, 1971). The value of this definition has been confirmed recently by studies showing that daydreaming is enrooted in a specific large-scale neurobiological network (Mason et al., 2007; Christoff et al., 2009) known as the Default Mode Network (DMN). The DMN is a neural network that is highly active during rest and less active, if not deactivated, during intense task engagement (Raichle et al., 2001). This network has been associated with a list of mental functions that are characterized by an internal focus, among which daydreaming seems to play a major role. Mason et al. (2007) have shown that when participants' minds drifted away from a well-practiced task, high levels of DMN activation were observed. Interestingly, the activation levels of DMN hub areas were correlated with a well-established self-report measure of daydreaming, the Daydreaming Frequency Scale (DDFS, Singer and Antrobus, 1970).

As mental baseline, daydreaming is a frequent phenomenon. Estimates suggest that we spend 30–50% of our mental activity during waking hours in thoughts that are neither related to what we are doing at that moment nor to the immediate surrounding environment (Klinger and Cox, 1987/88; Killingsworth and Gilbert, 2010; Franklin et al., 2013). In light of this ubiquity, it would be hard to believe that SGT does not serve specific functions, be it adaptive or maladaptive (Klinger, 1996, 2013). Although rarely studied until recently, the benefits of SGT are increasingly being reported in regard to different domains (Mooneyham and Schooler, 2013), for instance creative thinking (Baird et al., 2012), autobiographical planning (Smallwood et al., 2011), and delaying gratification (Smallwood et al., 2013). Nevertheless, several costs of SGT have been documented as well. Daydreaming, and specifically the mindwandering subtype, has been shown to detrimentally impact on reading comprehension (Franklin et al., 2011), sustained attention (Smallwood et al., 2004), and working memory (McVay and Kane, 2012).

In line with these findings, daydreaming, especially if characterized by negative cognitions, has been associated with symptoms of psychopathology, such as depression, schizophrenia, anxiety, and dissociation (Klinger et al., 2009; Andrews-Hanna et al., in press). What also confirms the potentially toxic role of daydreaming is that its neurobiological substrate has been consistently found to be affected in major psychopathology, such as schizophrenia and depression (Whitfield-Gabrieli and Ford, 2012).

Depression is an important context within which to investigate the clinical impact of daydreaming, as this disorder involves spending much time in inactivity, after which higher levels of depressed mood and lower levels of mastery and pleasure are shown (Martinsen, 2008). In line with this, many studies have indeed reported a clear and direct relation between daydreaming and depressive symptoms (Golding and Singer, 1983; Stawarczyk et al., 2011; Epel et al., 2013). For instance, Giambra and Traynor (1978) have shown that the frequency of and the tendency to be absorbed by daydreaming, especially if negatively valenced, correlated with three different measures of depression. Recently, Meyer et al. (2011) confirmed this finding, reporting that the tendency to engage in daydreaming was predicted by both the severity of current depressive symptoms and the likelihood of former depressive episodes. Furthermore, in a laboratory setting, individuals with subclinical levels of depression exhibited more accessible periods of mindwandering while encoding verbal material, greater attentional control failures, and higher physiological response than euthymic individuals (Smallwood et al., 2007).

Nevertheless, other studies did not support this link between depression and daydreaming in the same clear way, but proposed a more specific relation. Deng et al. (2012) reported that levels of depressive symptoms correlated only with the rate of episodes of mindwandering that occurred without the participant's awareness of being off task (e.g., l “zoned out”), but not with those episodes of which a participant was aware (e.g., “tuned out”). Moreover, Marchetti et al. (2012b) showed that individuals' levels of depressive symptoms were not correlated with mindwandering, but the former moderated the latter in predicting the accessibility of negative thinking. In keeping with this result, Smallwood et al. (2004/05) also reported that the rate of being off task correlated with individuals' mean scores of depression, but only in high ruminators and not in low ruminators.

This inconsistency in findings highlights the need to clarify the mechanism(s) through which daydreaming can lead to negative outcomes. Shedding light on the underlying process could indeed help understand what conditions increase the likelihood of negative outcomes related to daydreaming. Recently, Marchetti et al. (under review) proposed a comprehensive model that could explain the depressogenic role of daydreaming via contribution of multiple cognitive risk factors, such as rumination. In keeping with this, Marchetti et al. (2013) demonstrated in a laboratory setting that higher levels of internal focus during resting state predicted increased levels of state rumination that, in turn, explained a temporary worsening in mood. This model specifically held in individuals at-risk for depression. The authors speculated that being internally focused during rest could facilitate the emergence of self-related material that is the ideal condition for rumination to occur (Nolen-Hoeksema et al., 2008). Rumination, in turn, has been consistently found to enhance depressive symptoms (Aldao et al., 2010). Literature consistently reports that during SGT external information is processed to a lesser extent (Smilek et al., 2010; Barron et al., 2011), and the train of thoughts is largely insulated (Smallwood et al., 2012). Such reduced processing of external distractions could augment repetitive thinking (Nolen-Hoeksema et al., 2008).

In the current study, we aimed to test the indirect effect hypothesis that: (a) during daydreaming, self-related material would be significantly present in individuals' awareness; (b) being self-focused during SGT would spur ruminative processing of the emerged material; (c) a rigid and judgmental evaluation of internal material would lead to depressive symptoms. Given the inconsistent findings mentioned above, we did not make any *a priori* hypothesis about a direct association between daydreaming and depressive symptoms. Our study contributes to this research field in different ways. Importantly, by testing this model, we can further specify the mechanisms through which daydreaming is toxic and detrimental for mental health. Moreover, by relying on self-report questionnaires, our study may complement previous research that, although methodologically rigorous, suffers from suboptimal ecological validity, such as fMRI or specific laboratory contexts.

Therefore, we administered several trait questionnaires to measure individual levels of daydreaming (vs. mindfulness), self-focus, rumination, and depressive symptoms. Importantly, the scale we adopted as a measure for spontaneous cognitions, the DDFS, was previously used in both neurobiological and behavioral studies that confirmed its solid relation with the DMN (Mason et al., 2007) and rest-related phenomena, such as mindwandering (Mrazek et al., 2012; Stawarczyk et al., 2012). Moreover, in order to evaluate the specific role of daydreaming in predicting depressive outcomes, we controlled for dispositional level of mindfulness. Mindfulness has been defined in different ways, but here we focused on the perspective that defines mindfulness as sustained non-distraction from here and now (Brown and Ryan, 2003). By partialling it out, we could establish the specific role of daydreaming above and beyond the potential confound of mindfulness.

## Materials and methods

### Participants

We recruited 117 native Dutch-speaking students at Ghent University (mean age 21.51 ± 3.04, range: 20–46, F: 116 and M: 1^1^). This study was approved by the Ethical Committee of the faculty of Psychology and Education of Ghent University.

### Design

The questionnaires were completed in a group setting. The order of the questionnaires was counterbalanced^2^.

### Materials

#### Daydreaming frequency scale (DDFS; Singer and Antrobus, 1970)

The DDFS is one of the scales forming the Imaginal Processes Inventory. It consists of 12 items used to assess the frequency of daydreaming. Respondents rate each item on a 5-point Likert scale. Previous studies have reported good to excellent psychometric properties (Singer and Antrobus, 1970). For instance, both the English and the French version have been found to be unifactorial with substantial loading of each item (Singer and Antrobus, 1970; Stawarczyk et al., 2012). The instrument also has excellent 6–8 years test-retest reliability (*r* = 0.76) and internal consistency (Cronbach's α = 0.91) (Giambra, 1980). In the current study, the original 12 items were translated from English to Dutch independently by two native Dutch speakers with excellent knowledge of academic English. Importantly, one of the translators was one of the authors of this study (Ernst H. W. Koster), whereas the other translator was not involved in this research. Discrepancies between these two versions were discussed until a satisfactory version was found. In this study, excellent internal consistency was observed (Cronbach's α = 0.91).

#### Self-reflection and insight scale (SRIS; Grant et al., 2002)

The SRIS is a 20-item self-report questionnaire, consisting of two independent subscales, the Self-Reflection subscale (SRIS-SR) and the Insight subscale (SRIS-IN). The SRIS-SR scale includes 12 items and measures the tendency to self-focus, that is to think about one's own thoughts, actions, and feelings and evaluate them. The SRIS-IN consists of 8 items that assess clarity of experience and self-knowledge. Each item is measured on a 6-point Likert scale ranging from 1 (strongly disagree) to 6 (strongly agree). The SRIS has high internal consistency and internal validity (Grant et al., 2002; Roberts and Stark, 2008). The Dutch version of the questionnaire had good psychometric properties (Sauter et al., 2010) and in the current sample, the SRIS-SR and SRIS-IN showed excellent internal consistency (Cronbach's α = 0.94 for SRIS-SR; α = 0.82 for SRIS-IN).

#### Ruminative responses scale (RRS; Treynor et al., 2003)

The RRS is a 22-item self-report questionnaire that measures habitual tendency to ruminate and consists of items that describe responses to depressed mood that are focused on the self, symptoms, or consequences of this mood. Participants rate to what extent they usually engage in such responding using a 4-point Likert scale ranging from 1 (almost never) to 4 (almost always). Total RRS scores and subscale scores for reflection and depressive brooding were also calculated. The RRS has shown high reliability and validity and has good psychometric properties (Treynor et al., 2003). The Dutch version of the instrument also has good reliability and satisfactory validity (Raes et al., 2003). Internal consistency of the RRS and its subscales in the current study was good (Cronbach's α = 0.93 for the total score; α = 0.77 for the brooding subscale; α = 0.74 for the reflection subscale).

#### Beck depression inventory 2nd edition (BDI-II; Beck et al., 1996)

The BDI-II is a 21-item self-report questionnaire, which assesses the severity of affective, somatic and cognitive symptoms of depressive phenomenology. Individuals rate each symptom on a scale ranging from 0 to 3. The Dutch version of the BDI-II we used has acceptable reliability and validity (Van der Does, 2002). In our study, the BDI-II had excellent internal consistency (Cronbach's α = 0.85).

#### Mindful attention awareness scale (MAAS; Brown and Ryan, 2003)

The MAAS is a self-report 15-item questionnaire. Participants are required to rate each item on a 6-point Likert type scale, ranging from 1 (almost always) to 6 (almost never). The MAAS evaluates mindfulness as attention and awareness toward emotions, thoughts, sensations, and situations. Higher scores on the MAAS reflect higher levels of dispositional mindfulness. The Dutch translation of the MAAS was made available by the authors of the original instrument and in our study we found excellent internal consistency (Cronbach's α = 0.81).

### Data-analytic strategy

**Figure 1:**
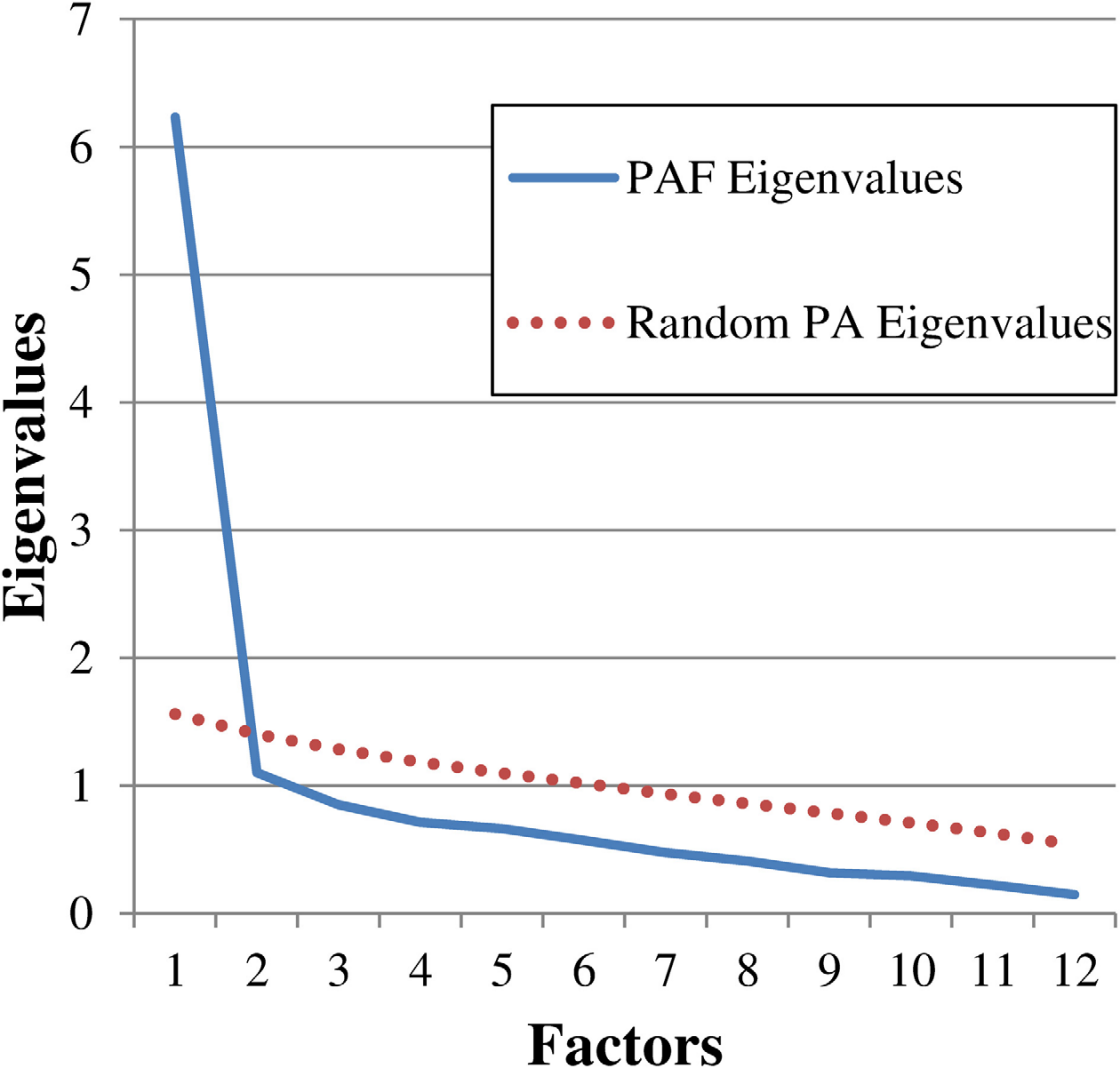
**Scree plot showing the eigenvalues derived from both the Principal Axis Factoring (PAF) and the Parallel Analysis (PA)**. PAF eigenvalues: 6.23, 1.10, 0.85, 0.71, 0.66, 0.57, 0.47, 0.41, 0.32, 0.29, 0.22, 0.15. Random PA eigenvalues (12 variables, *n* = 117, 1000 replications): 1.56, 1.40, 1.28, 1.18, 1.09, 1.01, 0.93, 0.85, 0.78, 0.70, 0.62, 0.53.

We first investigated the psychometric properties of the Dutch version of the DDFS. Initially, we checked the single item features and internal consistency through item analysis and Cronbach's alpha respectively. We then carried out an Exploratory Factor Analysis (EFA) on the Pearson's correlation matrix by means of Principal Axis Factoring (PAF) with oblique rotation (Oblimin) to highlight the factorial structure of the instrument. According to established guidelines (Zwick and Velicer, 1986), we retained the number of factors suggested by the scree plot (Cattell, 1966; see Figure 1), the Parallel Analysis (PA; Horn, 1965; see Figure 1), and the Minimum Average Partial Correlation statistic (MAP; Velicer, 1976). The analyses were carried out with IBM SPSS 19 and FACTOR 8.02 (Lorenzo-Seva and Ferrando, 2006).

We then checked the descriptive statistics and Pearson's correlations among all the variables measured in this study. Spearman's rank correlation coefficient was computed when necessary. Data were transformed to either obtain normally distributed variables or correct for outliers (*z* point > 3). No participants were excluded.

**Figure 2:**
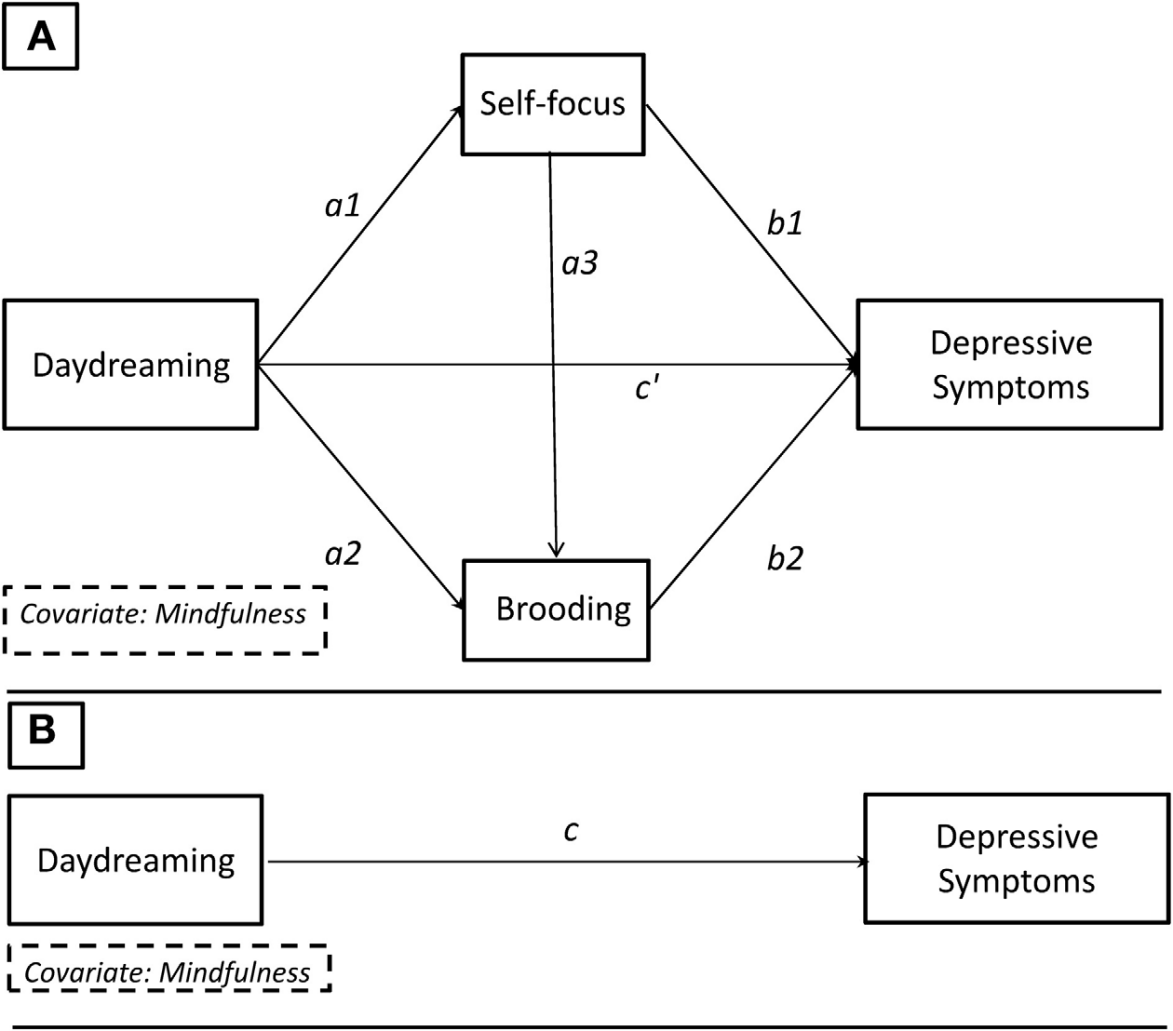
**Theoretical path diagram showing the multiple serial mediation model**. **(B)** Path c: total effect. **(A)** Path c′: direct effect. Path *a*_1_*a*_3_*b*_2_: specific indirect effect of interest.

According to our hypothesis (see Figure 2A), we first tested whether trait *daydreaming* could explain higher levels of *self-focus* (path *a*_1_), which in turn was expected to account for higher levels of *brooding* (path *a*_3_). The final output of this serial mediation model was individual levels of *depressive symptoms* (path *b*_2_). We did not have a specific hypothesis regarding either the total (path *c*) or the direct (path *c*′) effect of daydreaming with depressive symptoms. According to Mathieu and Mathieuand Taylor's (2006) guidelines, if the indirect effect was found significant, we could refer to this as an *indirect effect model* only if both the total and direct effect were null. If one or both of these two latter effects were found significant, we should speak of either *full* or *partial mediation model* (Preacher and Hayes, 2008). Given that daydreaming and mindfulness are thought to represent negatively related constructs, with the former capturing the tendency of the mind to drift away and the latter the tendency to be aware of the present moment (Deng et al., 2012; Mrazek et al., 2012), we always included the mindfulness as a covariate in all the models in which daydreaming was the focal predictor in order to establish its contribution above and beyond mindfulness^3^.

In our study, the total effect (path *c*) was computed as the regression coefficients of daydreaming and mindfulness predicting depressive symptoms, while none of the intervening variables were included in the model. According to the mediation theory (Hayes, 2013), it was possible to decompose the total effect in two main parts, that is the direct effect (path *c*′ with depressive symptoms being regressed on daydreaming, self-focus, brooding, and mindfulness) and the total indirect effect. The latter could, in turn, be decomposed into three specific indirect effects where daydreaming influenced depressive symptoms via: (i) self-focus (path *a*_1_*b*_1_); (ii) brooding (path *a*_2_*b*_2_); self-focus and brooding serially (path *a*_1_*a*_3_*b*_2_). By definition, the sum of the direct effect and the total indirect effect equates to the total effect of daydreaming on depressive symptoms.

To test the significance of both the total and specific indirect effects, we adopted the bootstrapping approach (Preacher and Hayes, 2008). Compared with the causal steps approach (Baron and Kenny, 1986) or the Sobel test (Sobel, 1982), bootstrapping is considered the most powerful approach, to be free from unrealistic assumptions (i.e., normal distribution of the indirect effect), and to have better control on Type I error (Hayes, 2009, 2013). In line with Preacher and Hayes' recommendations (2008), to test the significance of the indirect effects, we estimated 10,000 bootstrap bias-corrected 95% confidence intervals (CIs), and if they did not contain zero they were considered significant. The core hypothesis we tested was the model whereby daydreaming influences depressive symptoms via self-focus and brooding serially (path *a*_1_*a*_3_*b*_2_). However, in order to rule out alternative paths belonging to the same statistical model, we also estimated the other specific indirect effects (Hayes, 2013), where the intervening variables were introduced one at a time, that is either path *a*_1_*b*_1_ or path *a*_2_*b*_2_. In order to clarify the direction of the indirect effects, we also estimated each single path (e.g., *a*_1_, *a*_2_, *a*_3_, *b*_1_, and *b*_2_) using an ordinary least squares regression. Finally, in adherence with Mathieu and Taylor's guidelines (2006), we tested both the direct (path *c*′) and the total effect (path *c*).

Given that our study was purely cross-sectional, we tried to rule out two other serial indirect effects that would work against our main hypothesis. In the first alternative model, we substituted brooding with the reflection subscale of the RRS as the second intervening variable. Although both reflection and brooding are essentially forms of self-focus and self-referential thinking, reflection is known not to lead to negative outcomes, such as depressive symptoms (Nolen-Hoeksema et al., 2008). We thus expected the model, in which daydreaming influences depressive symptoms via self-focus and reflection, not to be significant. The second alternative model proposes that self-focus leads to increased daydreaming, which in turn could explain depressive brooding and depressive symptoms. Self-focus induction indeed has been reported to influence the tendency of the mind to drift away from external reality toward the inner mental world (Smallwood et al., 2011). However, it is difficult to explain how and why daydreaming, after being purged of its self-related component, could lead to rumination. Therefore, we anticipated this model would not be significant either.

All the analyses were carried out with IBM SPSS 19 and the macro PROCESS 2.03 (Hayes, 2013).

## Results

### Preliminary analysis: psychometric properties of the daydreaming frequency scale (DDFS)

We evaluated the psychometric properties of the Dutch version of the DDFS on the total sample. The inter-item correlation matrix showed that all the DDFS items were positively correlated, mean *r* = 0.47 (range: 0.17–0.84), as well as the mean-corrected item-total correlation was *r* = 0.65 (range: 0.51–0.79). Cronbach's alpha revealed excellent internal consistency (α = 0.91), which was not improved by item deletion. Before conducting the PAF on the 12 items, we checked the assumptions through the Kaiser-Meyer-Olkin criterion (KMO = 0.91) and the Bartlett's test of sphericity [χ^2^_(66)_ = 773.28, *p* < 0.001], which highlighted sufficient sample size and data quality (Gorsuch, 1983).

The scree plot inspection (see Figure 1), the Parallel Analysis (PA; Figure 1), and the Minimum Average Partial Correlation test (MAP, average partial correlation = 0.331) strongly supported the one-factor solution, which explained 51.93% of the variance in the unrotated matrix. All the items loaded on the factor substantively, that is = 0.54 (range: 0.54–0.84). According to Stevens (2002), for sample sizes of 100 subjects, only loadings greater than.51 should be interpreted.

In sum, we confirmed that the Dutch version of the DDFS is unifactorial and all the items significantly represent the underlying factor. We thus adopted the sum of the 12 DDFS items as our main variable in this study.

### Descriptive statistics and correlational analysis

Means, standard deviations, Cronbach's alphas and correlations between the questionnaire measures are reported in Table 1.

**Table 1:** **Means, standard deviations, Cronbach's alpha, and Pearson's correlations (*n* = 117)**.

	***M***	***SD***	***SK***	***K***	**Min–max**	**DDFS**	**SRIS-SR**^a^****	**SRIS-IN**	**RRS**^a^****	**Brooding**^a^****	**Reflection**	**BDI-II**^a^****	**MAAS**
DDFS	36.19	8.49	0.04	−0.48	18–58	(0.91)	0.21^*^	−0.21^*^	0.24^**^	0.21^*^	0.17^s^	0.12	−0.25^**^
SRIS-SR	51.39	9.91	−0.70	0.00	23–68		(0.94)	−0.12	0.42^***^	0.40^***^	0.55^s^^***^	0.15	0.13
SRIS-IN	31.66	5.9	−0.41	0.02	15–44			(0.82)	−0.21^*^	−0.18	−0.06^s^	−0.28^**^	0.39^***^
RRS	39.41	10.92	1.01	1.28	22–79				(0.93)	0.88^***^	0.73^s^^***^	0.36^***^	−0.01
Brooding	9.48	3.04	0.84	0.73	5–19					(0.77)	0.56^s^^***^	0.38^***^	0.01
Reflection	7.98	2.6	1.14	0.96	5–16						(0.74)	0.13^s^	0.07^s^
BDI-II	8.56	6.46	1.41	2.47	0–34							(0.85)	−0.25^**^
MAAS	4.17	0.59	−0.38	0.14	2.53–5.47								(0.81)

* p < 0.05;

** p < 0.01;

*** p < 0.001.

The values between parentheses are Cronbach's alphas. SK, skewness; K, kurtosis; DDFS, Daydreaming Frequency Scale; SRIS-SR, Self-reflection scale of the Self-reflection and Insight Scale; SRIS-IN, Insight scale of the Self-reflection and Insight Scale; RRS, Ruminative Response Scale—total score; BDI-II, Beck Depression Inventory 2nd Edition; MAAS, Mindful Attention Awareness Scale.

a Data transformed to either obtain normally distributed variables or correct for outliers (z point > 3).

s Spearman's rank correlation coefficient.

In line with our hypothesis, daydreaming frequency was positively correlated with self-focus measured with the self-reflection scale (SRIS-SF). Daydreaming was also correlated with rumination, and specifically with depressive brooding, but not with reflection. In line with a previous study and current theoretical perspectives (Marchetti et al., 2013, under review), daydreaming seems to be a phenomenon during which evaluative and judgmental self-referential thinking occurs. This was also supported by the negative relationship between the insight scale (SRIS-IN) and daydreaming. Daydreaming seems not to be beneficial with regard to the clarity of reflection and self-understanding; on the contrary, it may impair these processes. In sum, although daydreaming is focused on the daydreamer's narrative self (e.g., action, feelings, past events, etc.), it is not associated with any immediate beneficial outcome, rather it is the ideal condition for detrimental ruminative self-focus to occur. Moreover, daydreaming was independent from depressive symptoms, whereas it was negatively correlated with being aware at the present moment. It is noteworthy that this modest negative relation between daydreaming and mindfulness has been reported previously in other studies (i.e., *r* = −0.237; Mrazek et al., 2012). Importantly, not only does this result confirm a previous finding (Mrazek et al., 2012), but also it provides information about the divergent validity of the Dutch version of the DDFS.

### Mediation analysis

**Figure 3:**
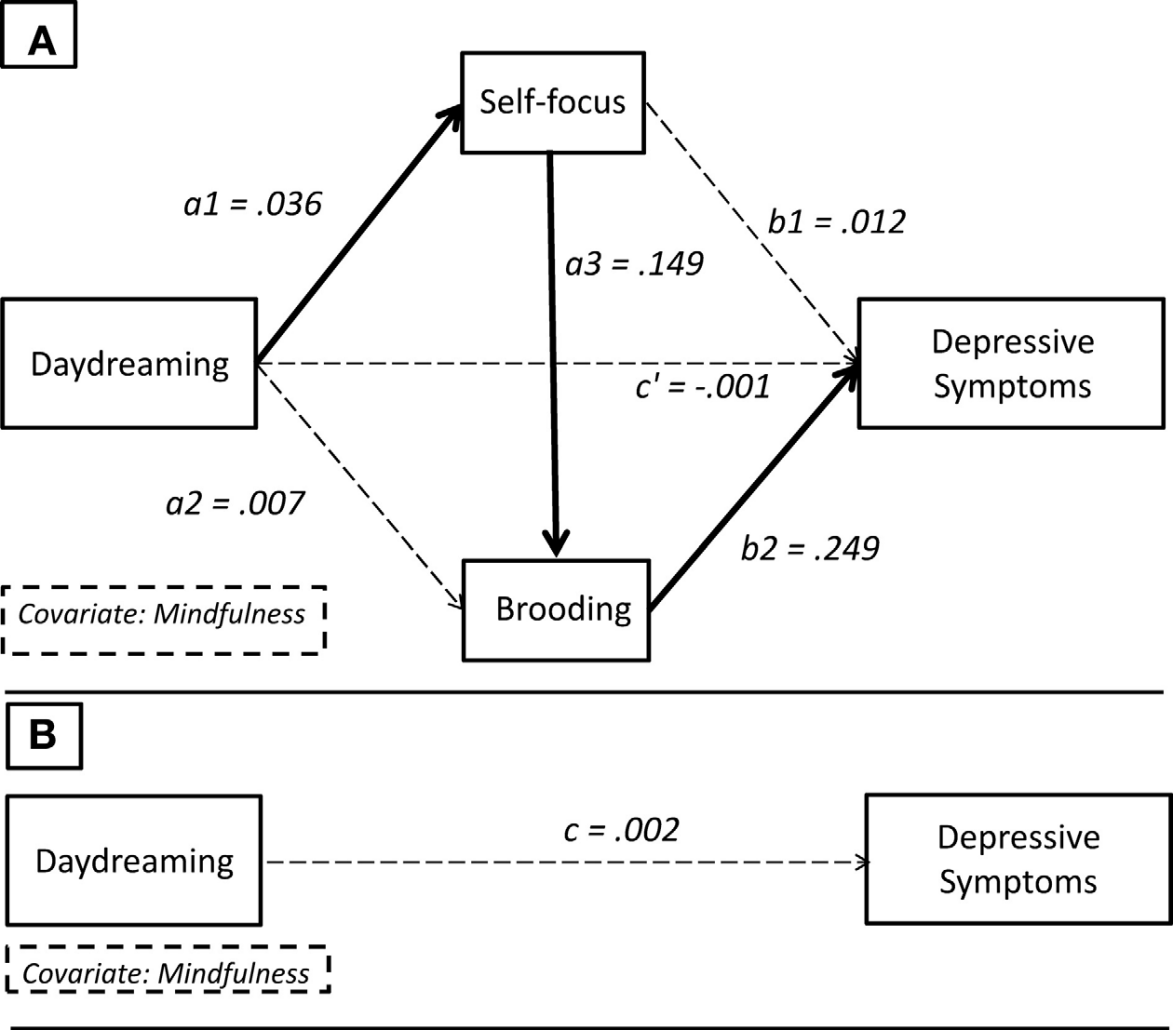
**Statistical diagram with path coefficients**. **(B)** Total effect: *c* = 0.002. **(A)** Direct effect: *c*′ = −0.001. Specific indirect effect of interest: *a*_1_*a*_3_*b* = 0.036(0.149)0.249 ≈ 0.0014. Bold paths are statistically significant, while dashed paths are not significant.

In accordance with Mathieu and Taylor's guidelines (2006), we first tested the significance of the main indirect effect of interest, namely that daydreaming would explain the levels of depressive symptoms via self-focus and brooding levels serially (path *a*_1_*a*_3_*b*_2_; Figure 2). Table 2 shows that this three-step indirect effect was indeed statistically significant (path *a*_1_*a*_3_*b*_2_ = 0.0014; boot 95% CI LL = 0.0004, boot 95% CI UL = 0.0036). Moreover, all the single paths of this effect were in the expected direction (Table 3). Indeed, daydreaming positively predicted self-focus (path *a*_1_ = 0.036), which, in turn, positively predicted brooding (path *a*_3_ = 0.149). Finally, brooding positively predicted depressive symptoms (path *b*_2_ = 0.249). It is noteworthy that the simpler alternative indirect paths were both not significant. The indirect effect whereby daydreaming influences depressive symptoms only via self-focus failed to reach statistical significance (path *a*_1_*b*_1_ = 0.0005; boot 95% CI LL = −0.0012, boot 95% CI UL = 0.0029). So did the other alternative path, whereby daydreaming predicts depressive symptoms via brooding levels (path *a*_2_*b*_2_ = 0.0019; boot 95% CI LL = −0.0006, boot 95% CI UL = 0.0057). In sum, the three-step indirect effect was the only statistically significant effect, and, despite that it consisted of two intervening variables, it was parsimonious too, in that simpler models did not explain the data satisfactorily (see Figure 3).

**Table 2:** **Specific and total indirect effects' unstandardized coefficients, standard error, and 95% bias-corrected confidence intervals^a^ (***n*** = 117)**.

**Path**	**Indirect effect coefficient**	**Boot *SE***	**Boot LL CI 95%**	**Boot UL CI 95%**
*a*_1_*b*_1_	0.0005	0.0010	−0.0012	0.0029
*a*_2_*b*_2_	0.0019	0.0015	−0.0006	0.0057
*a*_1_*a*_3_*b*_2_	0.0014	0.0008	0.0004	0.0036
Total indirect effect	0.0037	0.0019	0.0007	0.0082

a_*1*_b_*1*_, Daydreaming → Self-focus → Depressive symptoms; a_*2*_b_*2*_, Daydreaming → Brooding → Depressive symptoms; a_*1*_a_*3*_b*_2_*, Daydreaming → Self-focus → Brooding → Depressive symptoms.

a Mindfulness score (MAAS) was included as covariate.

**Table 3:** **Total (*c*) and direct (*c*′) effects' unstandardized coefficients, unstandardized path coefficients, standard errors, and *p*-values (*n* = 117)**.

**Antecedents**	**Path**	**Consequent**														
		**Self-focus (SRIS-SR)**			**Path**	**Brooding**			**Path**	**Depressive symptoms (BDI-II)**			**Path**	**Depressive symptoms (BDI-II)**		
		**Coefficient**	***SE***	***p*-value**		**Coefficient**	***SE***	***p*-value**		**Coefficient**	***SE***	***p*-value**		**Coefficient**	***SE***	***p*-value**
Daydreaming (DDFS)	*a*_1_	0.0366	0.0132	<0.01	*a*_2_	0.0076	0.0051	*ns*	*c*′	−0.0012	0.0034	*ns*	*c*	0.0024	0.0035	*ns*
Self-focus (SRIS-SR)		–	–	–	*a*_3_	0.1492	0.0352	<0.001	*b*_1_	0.0127	0.0252	*ns*		–	–	–
Brooding		–	–	–		–	–	–	*b*_2_	0.2493	0.0625	<0.001		–	–	–
Mindfulness (MAAS)		0.4057	0.1911	<0.05		−0.0068	0.0733	ns		−0.1472	0.0487	<0.005		−0.1286	0.0515	<0.01
(Constant)		−6.0386	1.0382	<0.001		3.2459	0.4450	<0.001		0.8184	0.3588	<0.001		1.3262	0.28	<0.001
		*R*^2^ = 0.079,				*R*^2^ = 0.180,				*R*^2^ = 0.210,				*R*^2^ = 0.066,		
		*F*_(2, 114)_ = 4.869, *p* < 0.01				*F*_(3, 113)_ = 8.249, *p* < 0.001				*F*_(4, 112)_ = 7.458, *p* < 0.001				*F*_(2, 114)_ = 4.044, *p* < 0.05		

All the coefficients are unstandardized.

We then tested both the total (path *c*) and the direct (path *c*′) effect using the OLS regression approach. Table 3 shows that both unstandardized coefficients were not significant. We can thus conclude that the hypothesized serial (indirect) effect model was supported and that daydreaming seems to explain depressive symptoms only via the contribution of both self-focus and depressive brooding.

### Additional analysis

We also explored two alternative models that were in contrast to our main hypothesis. We investigated whether the same three-step indirect effect was statistically significant, after removing brooding and including reflection as the second intervening variable. Given the unclear link between reflection and depression (Nolen-Hoeksema et al., 2008), we expected this model would not be significant. In keeping with this, Table 4 shows that this alternative model was in fact not statistically sound (path *a*_1_*a*_3_*b*_2_ = 0.0005; boot 95% CI LL = −0.0004, boot 95% CI UL = 0.0021).

**Table 4:** **Specific and total indirect effects' unstandardized coefficients, standard errors, and 95% bias-corrected confidence intervals^a^ (***n*** = 117)**.

**Path**	**Indirect effect coefficient**	**Boot *SE***	**Boot LL CI 95%**	**Boot UL CI 95%**
*a*_1_*b*_1_	0.0014	0.0013	−0.0005	0.0047
*a*_2_*b*_2_	0.0004	0.0007	−0.0003	0.0025
*a*_1_*a*_3_*b*_2_	0.0005	0.0006	−0.0004	0.0021
Total indirect effect	0.0022	0.0014	0.0001	0.0056

a*_1_*b*_1_*, Daydreaming → Self-focus → Depressive symptoms; a*_2_*b*_2_*, Daydreaming → Reflection → Depressive symptoms; a*_1_*a*_3_*b*_2_*, Daydreaming → Self-focus → Reflection → Depressive symptoms.

a Mindfulness score (MAAS) was included as covariate.

By capitalizing on the literature (Smallwood et al., 2011), we also put forward that self-focus could lead to a habitual tendency to daydream that in turn could explain depressive symptoms via the contribution of brooding. In contrast, our main hypothesis argued that daydreaming would lead to depressive brooding only via the self-referential focus of task-free mental activity. Because of this, we expected that this alternative model would not reach significance. Table 5 shows indeed that self-focus fails to explain depressive symptoms via daydreaming and brooding serially (path *a*_1_*a*_3_*b*_2_ = 0.0028; boot 95% CI LL = −0.0003, boot 95% CI UL = 0.0113).

**Table 5:** **Specific and total indirect effects' unstandardized coefficients, standard errors, and 95% bias-corrected confidence intervals (***n*** = 117)**.

**Path**	**Indirect effect coefficient**	**Boot *SE***	**Boot LL CI 95%**	**Boot UL CI 95%**
*a*_1_*b*_1_	0.0025	0.0047	−0.0054	0.0139
*a*_2_*b*_2_	0.0373	0.0141	0.0143	0.0695
*a*_1_*a*_3_*b*_2_	0.0028	0.0027	−0.0003	0.0113
Total indirect effect	0.0426	0.0151	0.0169	0.0762

a_*1*_b*_1_*, Self-focus → Daydreaming → Depressive symptoms; a*_2_*b*_2_*, Self-focus → Brooding → Depressive symptoms; a*_1_*a*_3_*b_*2*_, Self-focus → Daydreaming → Brooding → Depressive symptoms.

## Discussion

SGT and mental phenomena that are based on it, such as daydreaming, are increasingly attracting scholars' attention (Klinger, 1971, 1990, 2009, 2013; Smallwood and Schooler, 2006; Andrews-Hanna et al., in press; Smallwood, 2013) given their ubiquitous impact on mental life. Interestingly, daydreaming has been associated with increased depressive symptoms and negative cognitions (Smallwood et al., 2007; Meyer et al., 2011), although findings are mixed. Here we sought to examine some of the pathways that could potentially explain why and how daydreaming leads to depressive outcomes and, in turn, account for the inconsistency reported in the literature.

In our study, we found that levels of daydreaming and depressive symptoms were statistically independent. However, according to the previous studies and a recent theoretical framework (Marchetti et al., 2013, under review), daydreaming did predict depressive outcomes, but only to the extent to which self-focus and brooding were involved too. In other words, during SGT, our attention tends to be focused on internal scenarios related to our self and self-related goals (Northoff et al., 2006; Klinger, 2009; Diaz et al., 2013). This enhances the chance of ruminating on the (lack of) progress in salient goal-striving (Koster et al., 2011; Klinger, 2013). Unfortunately, such a passive and self-critical evaluation has consistently been reported to be depressogenic (Aldao et al., 2010). It is also noteworthy that, in line with these results, daydreaming was negatively correlated with both mindfulness and the clarity of self-knowledge. That is, people who reported experiencing daydreaming generally did not benefit from being aware of the present moment nor did being self-focused lead them to a better understanding of themselves.

On the one hand, these findings are important because they convey a plausible homological model suggesting possible directional links between crucial constructs in depression, such as daydreaming, self-focus, and brooding. It is also noteworthy that the proposed model could bridge the gap between cognitive and neurobiological science, in that the hypothesis tested in this study is compatible with evidence derived from both research fields. As mental baseline, daydreaming is considered the quintessential outcome of the DMN (Mason et al., 2007), while self-focus has been robustly associated with a specific DMN subnetwork, Cortical Midline Structures (CMS; Northoff et al., 2006). Unsurprisingly, both rumination and clinical depressive status, too, have been linked with higher levels of DMN functional connectivity during resting state (Greicius et al., 2007; Berman et al., 2011; Zhu et al., 2012). Therefore, the model tested in this study holds promise for guiding future neuroimaging studies where trait rumination could be associated with specific dynamics of neural activation of DMN-related brain regions (i.e., Granger causality test; Hamilton et al., 2011).

On the other hand, we believe that our study not only replicated previous findings, but also complements these research lines that may suffer from suboptimal ecological validity. Both fMRI investigations and experimental studies usually require individuals to rest in conditions that are far from those usually experienced. For instance, recent methodological studies highlight the detrimental impact of the scanner background noise on the neural activation of DMN and resting state (Gaab et al., 2008; Hommel et al., 2012; Rondinoni et al., 2013). On the contrary, in our study we did not impose any artificial condition, but we simply investigated stable and long-term dispositions through self-report. By doing so, we may have been able to track specific mechanisms that are more likely to mirror what happens in everyday life, although future experience sampling studies are warranted.

This study has several limitations that we want to acknowledge. First, the research design is totally self-report based and methodologically cross-sectional, so that no cause-effect claims can be made. Nevertheless, to partially mitigate this flaw, we ruled out four alternative models. Not only did this support our hypothesis, but it also confirmed the validity of the model, in that no redundant variable was detected. Apparently, both self-focus and maladaptive rumination were necessary components for daydreaming to impact on depressive symptoms. However, in order to be able to further validate the model, behavioral high-risk longitudinal designs are warranted (Alloy et al., 1999). Second, we acquired information only about the frequency of daydreaming and not on its specific content. According to recent perspectives on SGT (Smallwood and Andrews-Hanna, 2013), a factor implicated in the impacting of daydreaming on well-being is its specific content, either positive vs. negative, or past- vs. future-oriented (Klinger et al., 2009; Ruby et al., 2013). Accordingly, future studies should include additional significant variables in order to better specify under which circumstances daydreaming leads to depressive outcomes and, most importantly, when this is not the case. Third, according to our hypothesis aiming at clarifying the role of daydreaming in depression, we did not take into account the possibly positive effects of SGT. For instance, previous research has indeed reported that DDFS is positively correlated with creative thinking (Baird et al., 2012) and neurobiological evidence suggests that SGT could facilitate social cognition (Schilbach et al., 2008, 2012). So, we cannot exclude that possible positive effects due to SGT did go unnoticed in our study.

In sum, the clinical importance of resting state and rest-related phenomena is increasingly being stressed by both researchers and clinicians (Rosner et al., 2004; Whitfield-Gabrieli and Ford, 2012), with different models being proposed (i.e., Marchetti et al., 2012a, under review; Andrews-Hanna et al., in press). In our study, we confirmed a plausible mechanism recently proposed by Marchetti et al. (2013), whereby daydreaming is supposed to impact on depression via contribution of both self-focus and rumination. However, we are not claiming here that daydreaming is negative *per se*. On the contrary, we have been able to clarify a specific mechanism where self-focus seems to be pivotal (Green, 1923). This also implies that, in individuals with a different style, daydreaming might have different effects, such as in those who tend to be more other-focused than purely self-focused during free thinking (Mar et al., 2012; Marchetti and Koster, 2014).

In conclusion, daydreaming is a very fluid and complex mental activity. Theoretical and empirical efforts are necessary to highlight both the negative and positive consequences of such a pervasive phenomenon that occupies a vast part of our mental life.

### Conflict of interest statement

The authors declare that the research was conducted in the absence of any commercial or financial relationships that could be construed as a potential conflict of interest.

## Footnotes

^1^All the analyses reported in this study did not substantially change after excluding the only male participant.

^2^Another trait questionnaire of self-focus, the Private Self-Consciousness Scale (PrSCS, Fenigstein et al., 1975), was also administered. The analysis did not change substantially when the PrSCS was used. For sake of brevity, these results were not reported.

^3^All the analyses reported did not substantially change after excluding MAAS as covariate.
